# Normal Iron Homeostasis Requires the Transporter SLC48A1 for Efficient Heme-Iron Recycling in Mammals

**DOI:** 10.3389/fgeed.2020.00008

**Published:** 2020-10-20

**Authors:** William R. Simmons, Lily Wain, Joseph Toker, Jaya Jagadeesh, Lisa J. Garrett, Rini H. Pek, Iqbal Hamza, David M. Bodine

**Affiliations:** ^1^Hematopoiesis Section, Genetics and Molecular Biology Branch, National Human Genome Research Institute (NHGRI), Bethesda, MD, United States; ^2^National Human Genome Research Institute (NHGRI) Embryonic Stem Cell and Transgenic Mouse Core Facility, Bethesda, MD, United States; ^3^Department of Animal & Avian Sciences, University of Maryland, College Park, MD, United States

**Keywords:** gene editing (CRISPR-Cas9), erythrocyte phagocytosis, erythropoiesis, mouse model, anemia

## Abstract

In mammals over 65% of the total body iron is located within erythrocytes in the heme moieties of hemoglobin. Iron homeostasis requires iron absorbed from the diet by the gut as well as recycling of iron after the destruction of senescent erythrocytes. Senescent erythrocytes are engulfed by reticuloendothelial system macrophages where hemoglobin is broken down in the lysosomes, releasing heme for iron recovery in the cytoplasm. We recently showed that the SLC48A1 protein is responsible for transporting heme from the lysosome to the cytoplasm. CRISPR generated SLC48A1-deficient mice accumulate heme in their reticuloendothelial system macrophages as hemozoin crystals. Here we describe additional features of SLC48A1-deficient mice. We show that visible hemozoin first appears in the reticuloendothelial system macrophages of SLC48A1-deficient mice at 8 days of age, indicating the onset of erythrocyte recycling. Evaluation of normal and SLC48A1-deficient mice on iron-controlled diets show that SLC48A1-mediated iron recycling is equivalent to at least 10 parts per million of dietary iron. We propose that mutations in human *SLC48A1* could contribute to idiopathic iron disorders.

## Introduction

Hemoglobin within erythrocytes of an average adult human male contain about 2.5 g of iron, representing about 65% of the total body iron. The continuous production of new erythrocytes requires iron, some of which is absorbed from the diet by the gut. Absorbed iron is then bound to transferrin, which enters the circulation from where it is imported into developing orthochromic erythroblasts (Andrews, 2008; Giger and Kalfa, 2015). As erythrocytes senesce, the majority of iron is recovered from hemoglobin by reticuloendothelial system (RES) macrophages. After engulfing senescent erythrocytes, RES macrophages digest the hemoglobin, releasing heme. In the cytoplasm, the enzyme heme oxygenase processes heme to remove the iron atom, which is exported from the cell by ferroportin and bound by transferrin for passage to the bone marrow to produce new erythrocytes (Kong et al., 2013). In erythroblasts, imported transferrin-bound iron is subsequently incorporated into heme by a series of enzymes associated with the mitochondrial membrane (Chung et al., 2012; Kong et al., 2013). From there heme rapidly associates with nascent alpha and beta globin chains which are assembled into a heterotetrameric hemoglobin molecule (2 alpha chains with their associated heme molecules and 2 beta chains with their associated heme molecules).

The levels of heme synthesis and nascent globin chain translation are carefully regulated to allow efficient production of hemoglobin while avoiding toxicity due to excess heme (Ponka et al., 1998; Chung et al., 2012; Chen, 2014). There are contrasting views about how heme synthesis and hemoglobin assembly are coordinated. The original view is that the heme synthesis pathway and hemoglobin translation are co-regulated to synthesize exactly as much heme as is needed for the amount of globin chains present (Chen, 2014; Ponka et al., 2017). Genetic support for this view comes from the fact that mutations in the genes encoding the heme synthesis pathway enzymes are well-known and are causally related to a wide variety of hematologic disorders (Fontenay et al., 2006; Peoc'h et al., 2019). Similarly, heme has been shown to regulate the translation of erythroid proteins including globin chain translation through the action of Heme-regulated eIF2α kinase (HRI) (Keerthivasan et al., 2011; Zhang et al., 2019).

An emerging view posits that developing red cells also express heme transporters to keep the levels of heme and globin chains balanced. Early erythroblasts maintain stoichiometric amounts of heme and globin by expressing heme exporters to prevent free heme from exceeding globin levels, while reticulocytes, which have extruded their mitochondria, import heme needed for hemoglobin synthesis (Keerthivasan et al., 2011). Support for this view come from the discovery of a heme exporter, FLVCR, which has been shown to be expressed at high levels at the CFU-E stage before declining during terminal erythroid maturation (Quigley et al., 2004; Keel et al., 2008). While variants in *FLVCR* have been proposed to play a role in a wide variety of disorders, no causal relationship between an *FLVCR* variant and a disease has been discovered (Quigley et al., 2005; Gnana-Prakasam et al., 2011; Nieuwenhuizen et al., 2013). However, in animal models, deficiency of FLVCR causes a lethal anemia due to heme toxicity (Keel et al., 2008). A heme importer, HRG1 (encoded by the mammalian gene *SLC48A1*), was originally discovered in *C. elegans* (Rajagopal et al., 2008; White et al., 2013). Subsequently, the heme transport function of orthologs of HRG1 has been demonstrated in yeast models and for mammalian SLC48A1, in tissue culture models. Recently we described a mouse model of *SLC48A1* deficiency. *SLC48A1*-deficient mice are unable to transport heme from RES phagolysosomes into the cytoplasm. *SLC48A1*-deficient animals avoid heme toxicity because the lysosomal heme crystalizes into hemozoin, a supposedly inert form of heme. Prior to this finding, hemozoin had only been observed in the food vacuoles or lysosomes of blood-feeding parasites such as *Plasmodium* (Pek et al., 2019).

In this report we present additional phenotypic characterization of the SLC48A1 deficient animals. These include a complete analysis of the highly efficient gene editing at the *Slc48a1* locus (15 mutations in 36 founder animals; 41%) and the range of gene-edited mutations recovered. We also demonstrate that hemozoin begins to accumulate in RES macrophages 8 days after birth, which we propose correlates with the beginning of erythrocyte recycling in the mouse. Finally, we show that SLC48A1-deficient mice require more dietary iron to maintain erythropoiesis than littermate control animals.

## Methods

### Animals

All mice were housed in a 12 h light-dark cycle. Both male and female mice were used in all studies. No differences between the genders were observed. All animal protocols were approved by the NHGRI Animal Care and Use Committee and the Institutional Animal Care and Use Committee at the University of Maryland, College Park.

### Generation of HRG1^-/-^ Mice

Guide and Cas9 RNAs: Three guide RNAs (1 = 5′ TAGGGACGGTGGTCTACCGACAACCGG 3′; 2 = 5′ CGGTGGTCTACCGACAACCG 3′; 3 = 5′ AACCGGGGACTGCGGCGATG 3′) were purchased from Sage Laboratories 2033 Westport Center Drive, St Louis, MO. Cas 9 RNA was purchased from Trilink Biotechnologies, San Diego, CA. The guide RNA and Cas9 RNA were combined at a concentration of 5 ng/μl (each) in 10 mM Tris, 0.25 mM EDTA (pH 7.5) for pronuclear injection. Pronuclear injection was performed using standard procedures (Behringer et al., 2014). Briefly, fertilized eggs were collected from superovulated C57BL/6J females ~9 h after mating to 129/SvJ male mice (resulting animals are B6129F_1_). In a second set of experiments, fertilized eggs were collected from superovulated C57BL/6J females mated to C57BL/6N males (resulting animals are B6JB6NF_1_). In these experiments, Guide 1 RNA and Cas9 protein were combined at a concentration of 5 ng/μl (Guide 1) and 10 ng/ μl (Cas9) in 10 mM Tris, 0.25 mM EDTA (pH 7.5) to form a ribonuclear protein complex. All pronuclei were injected with a capillary needle with a 1–2 μm opening pulled with a Sutter P-1000 micropipette puller. The RNAs or ribonuclear protein were injected using a FemtoJet 4i (Eppendorf) with continuous flow estimated to deposit ~2 pl of solution. Injected eggs were surgically transferred to pseudo-pregnant BALB/cByJ x C57BL/6ByJ (CB6F_1_) recipient females.

DNA was obtained from founder (F_0_) animals by tail biopsy, amplified by PCR (Forward 5′-TGCACCTGTGACTCGGCG-3′ Reverse 5′-TAGGTCCCGCCACGTTCATAA-3′ and sequenced to determine the genotype. F_0_ animals carrying mutations were crossed to C57BL/6 animals and the resulting heterozygous F_1_ animals were either intercrossed to generate homozygous mutant animals or back crossed to C57BL/6 mice for propagation.

### Western Blot

Western Blots were performed as described previously (Pek et al., 2019). Spleen tissue was frozen in liquid nitrogen and ground using an ice cold mortar and pestle. The powdered spleen tissue was added to prep buffer (250 mM Sucrose, 1 mM EDTA, 10 mM Tris-HCl pH 7.4, 3X protease inhibitor cocktail) in a dounce homogenizer for further homogenization. Homogenates were centrifuged at 800 g for 10 min at 4°C, then at 100,000 g for 2 h at 4°C. The pellet was resuspended in lysis buffer (150 mM NaCl, 1 mM EDTA, 20 mM HEPES pH 7.4, 2% Triton-X, 3X protease inhibitor), sonicated and centrifuged at 11,000 g for 30 min at 4°C. The protein concentration of the supernatant was determined using the BCA assay (Pierce BCA Protein Assay Kit, Thermo Fisher Scientific, cat. Number 23225). Samples were mixed with SDS-loading buffer and separated on a 4–20% Criterion TGX Precast Midi Protein Gel (Bio-rad, cat. number 5671094). After transfer to a nitrocellulose membrane the proteins were cross-linked by UV treatment and stained with Ponceau S before incubation in blocking buffer (5% non-fat dry milk in 0.05% Tris-buffered saline-Tween 20) for 1 h at room temperature. Blots were then incubated overnight at 4°C in blocking buffer containing rabbit anti-SLC48A1 antibody (1:300 dilution). After three washes in 0.05% Tris-buffered saline-Tween 20, the blots were incubated 1 h with horseradish peroxidase (HRP)-conjugated goat anti-rabbit IgG secondary antibody (1:20000; Invitrogen cat. Number 31460) in blocking buffer. After the secondary antibody incubation, the membranes were washed five times with 0.05% Tris-buffered saline-Tween 20 and the signals visualized by using enhanced chemiluminescence (SuperSignal West Pico, Pierce) and detected using ChemiDoc Imaging Systems (Bio-Rad).

### Diet Study

The iron-controlled diets were custom ordered from Envigo (Madison, WI) and contained 5, 10, or 20 ppm iron, as measured by ICP-MS. Three breeding units consisting of M13 heterozygous littermates provided the *Slc48a1*^+/+^, *Slc48a1*^+/−^ and *Slc48a1*^−/−^ mice used in these studies. At least 3 l from each breeding unit were used.

The parental cages were manitained on an iron replete diet (400 ppm) and the date of birth of the litters was recorded. At least 3 l from each breeding unit were used. Tail biopsies were collected from the pups at 10 days of age (P10) for genotyping. At 15 days, when pups first begin to eat solid food, the food in the parental cages was switched to one of the three iron restricted diets (5, 10, or 20 ppm). On day 21, the pups were weaned into special cages containing the iron restricted diets and the 400 ppm diet was restored to the parental cages. Since mice derive ~25% of their nutrition from copography, the pups were placed in cages with wire bottoms to prevent feeding on feces. In addition, to prevent iron in the feces of wild type or heterozygors animals from rescuing SLC48A1 deficiency, the animals were segregated by genotype.

### CBC Studies

Fifty microliters of peripheral blood was collected at weaning (P21) and every week thereafter until week 14 by retro-orbital bleeding and the complete blood counts were determined. Retro-orbitally blood was drawn into heparinized capillary tubes (Fisher Scientific). Immediately after blood collection, it was ejected into EDTA tubes (Beckton Dickenson). Complete blood counts were performed using the Element HT5 Veterinary Hematology Analyzer (Heska). Data were aggregated in Microsoft Excel and analyzed using R Studio.

### Histology

Prenatal mice were harvested from timed C57BL/6 *SLC48a1*^+/−^ intercross matings at days E12.5 to birth. Post-natal animals were euthanized at days P0–21. The fetal liver, fetal spleen, postnatal spleen, and bone marrow were harvested and fixed in formalin. Paraffin-embedded tissue sections stained with hematoxylin and eosin by Histoserve (Rockville, MD).

## Results

### Generation of Mutations at the *Slc48a1* Locus

Initially we evaluated three different guide RNAs, which were injected into B6129F_1_ zygotes along with Cas9 protein. Guides 1 and 2 targeted overlapping regions of *Slc48a1* exon 1, while guide 3 targeted exon 2. At E14.5, embryos were analyzed for evidence of editing at the *Slc48a1* locus. Guide 1 generated 3/12 embryos with evidence of editing at the *Slc48a* locus, while Guide 2 generated 3/13 embryos with evidence of targeting. No animals with evidence of gene targeting were identified in the Guide 3 experiments.

**Figure 1:**
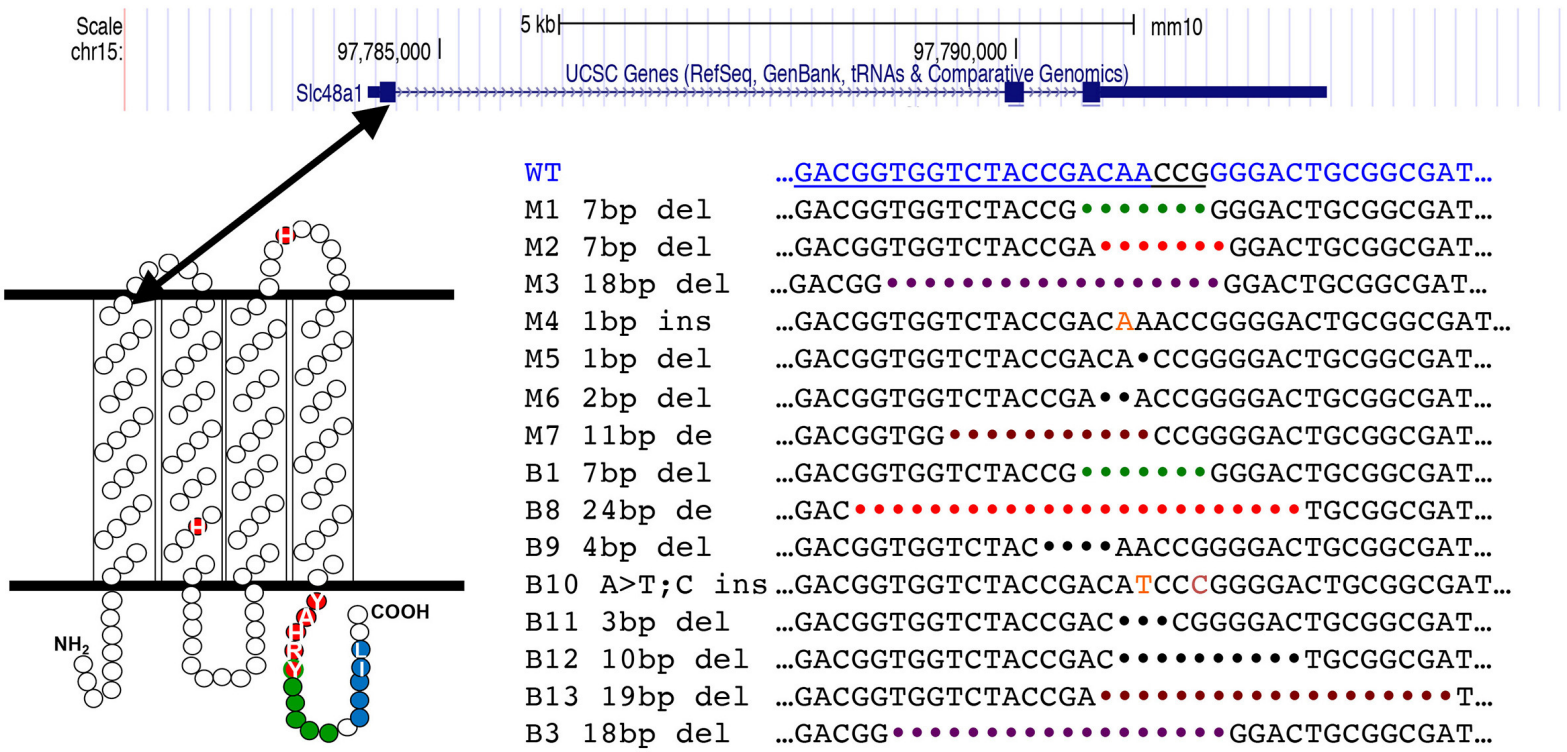
CRISPR generated mutations in the mouse *Slc48a1* gene. The top panel shows the mouse *Slc48A1* locus. Large boxes indicate coding sequence (exons), smaller boxes transcribed, non-coding sequence. The lines between exons represent introns. The first exon encodes the first transmembrane domain (lower left). The sequence of CRISPR Guide RNA 1 is shown in blue text at the top of the bottom right panel. The sequence of the mutations in the 15 transgenic mouse founder lines are shown in black text.

The SLC48A1 protein has four predicted membrane-spanning domains. Guide 1 targets the *Slc48a1* locus in the region of the first transmembrane domain of SLC48A1 (Figure 1), which we hypothesized would be more likely to cause loss-of-function mutations. Therefore, we repeated the Guide 1 injections and obtained 7/15 F_0_ (~47%) B6129F_1_ animals with edits in the *Slc48a1* locus. To generate mice on a more uniform background we performed a second round of injections with Guide 1/Cas9 ribonucleoprotein into B6BNF_1_ embryos. From a total of 21 F_0_ mice, we identified eight B6BNF_1_ F_0_ animals (~37%). In all cases, F_0_ animals were crossed to C57BL/6J mice for propagation. All analyses described were performed on animals backcrossed at least four generations to C57BL/6 before intercrossing.

Of the 15 mutations in the region targeted by Guide 1, we observed 13 deletions and two insertions. Five of the 15 gene edited sites were at or within 1 base of the PAM sequence. We observed two examples of different founder animals carrying identical mutations; a 7-base pair deletion (M1; B1) and an 18-base pair deletion (M3; B3).

### Characterization of *Slc48a1* Deficient Mice

The 12 lines with insertions or deletions had frame shifts beginning at ~amino acid 30 depending on the location of the editing. These frame shifts led to premature termination, truncating the SLC48A1 protein between amino acids 50 and 125. Homozygous mutant animals of all of the deletion/insertion lines had similar hemozoin accumulation in their spleens, marrow and liver. As we have previously reported, mice homozygous for *Slc48a1* frame shift mutations were born in a Mendelian ratio. Western blotting of spleen and liver tissue from homozygous mutant mice of the M6, M4, B10, and B13 lines showed a complete lack of SLC48A1 protein (Pek et al., 2019) (Supplementary Figure 1). Similarly, Slc48a1 mRNA was absent from homozygous mutant mice of multiple lines (Pek et al., 2019). The M3, B3, and B11 lines, all of which had in-frame deletions, were cryopreserved, but not evaluated.

SLC48A1-deficient mice fed the standard laboratory rodent diet (~400 ppm iron) had peripheral blood counts that were all within the normal range, including the red cell indices shown in Table 1. No differences were observed between male and female animals. As described previously, we did observe an ~15% increase in the size of the spleen in SLC48A1-deficient animals (Pek et al., 2019).

**Table 1:** Red cell indices of control and *Slc48a1* mutant mice.

**Genotype**	**RBC (10^**∧**^6)**	**Hemoglobin (g/dL)**	**HCT (%)**	**MCV (fL)**	***n* F/M**
+/+C57BL/6	9.052 (0.42)	15.2 (0.50)	46.50 (1.91)	48.76 (1.45)	9
+/+Littermate	10.186 (0.37)	15.54 (0.50)	44.22 (1.65)	43.48 (2.37)	5 3/2
+/*Slc48a1*Littermate	10.763 (0.28)	16.26 (1.25)	45.91 (3.41)	44.60 (2.28)	8 4/4
*Slc48a1*/*Slc48a1*Littermate	10.150 (0.63)	15.38 (0.98)	44.10 (2.80)	43.48 (1.84)	10 5/5

*Mean and Standard Deviation (parentheses) for each value are shown. The number of animals analyzed and the sex distribution are shown in the right column. We did not observe any differences in males and females so the data are pooled*.

### Accumulation of Hemozoin in SLC48A1-Deficient Mice

We have previously reported that the spleens, bone marrow, and livers of adult SLC48A1-deficient mice contained large amounts of black pigmented granules. Chemical extraction of this material followed by high resolution X-ray powder diffraction demonstrated that the dark pigment was identical to malarial hemozoin (Slater et al., 1991; Coronado et al., 2014; Pek et al., 2019). Immunohistochemistry, flow cytometry, and electron microscopy showed that the hemozoin crystals were present in RES macrophages (Pek et al., 2019).

**Figure 2:**
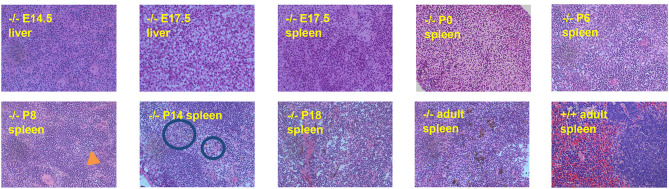
Hemozoin accumulation in SLC48A1 deficient mice. Hematopoietic tissues were collected at the indicated times, fixed, sectioned and stained with Hematoxylin and Eosin. Hemozoin appears as a black pigment, first visible on postnatal day 8 (P8; orange arrow). No hemozoin is observed prior to P8, in prenatal tissues or in wild type adult (8 week) spleen. Magnification 20X.

We hypothesized that heme concentrated in the lysosomes of RES macrophages would begin to crystalize as soon as the recycling of senescent red cells begins in in SLC48A1-deficient animals. To test this hypothesis, we examined the reticuloendothelial tissues of animals homozygous for the B13 mutation (19 base pair deletion; Figure 1) at different ages beginning prenatally and extending through birth (P0) to adulthood (>6 weeks). H and E staining of fetal liver and fetal spleen along with the spleen and bone marrow of newborn animals revealed no visible hemozoin, compared to the large amount of hemozoin visible in the spleens of adult animals (Figure 2). The first evidence of visible hemozoin was observed in the spleens of 8-day old animals (P8; Figure 2 and higher magnifications in Supplementary Figure 2). The hemozoin crystals at P8 were infrequent, but were shown to contain iron by Perl's staining (Supplementary Figure 2). Beyond P8, the number of visible hemozoin crystals increased steadily (Figure 2). We conclude that the recycling of senescent red blood cells occurs by at least 8 days of age.

### Deficiency of SLC48A1 Increases the Dietary Iron Requirement

**Figure 3:**
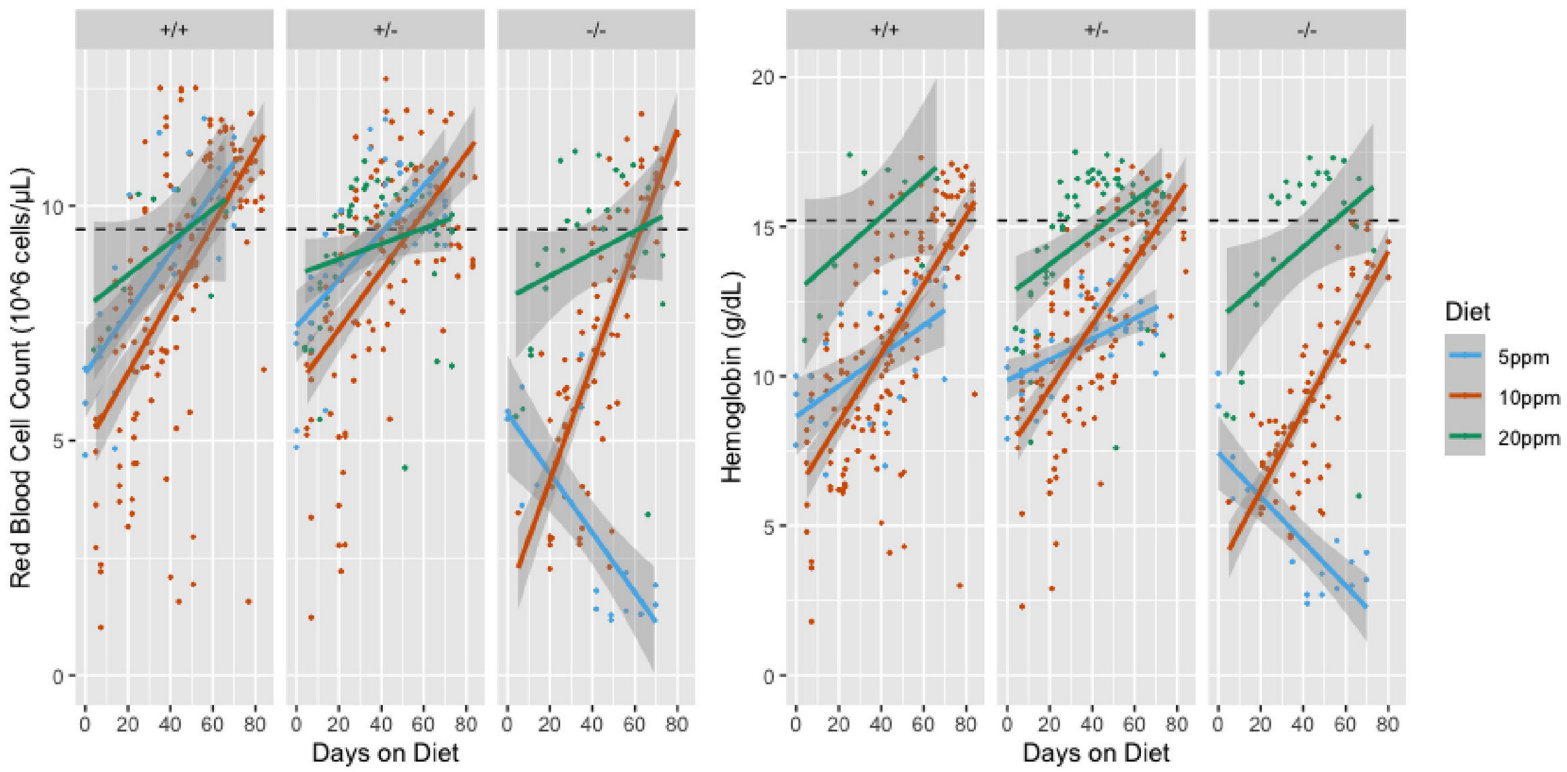
Red Blood Cell Counts (RBC; left panel) and Hemoglobin levels (Hb; right panel) of mice on iron restricted diets. The RBC or Hb values are shown on the Y-axis and the days on the iron restricted diet is shown on the X-axis. Genotypes are shown at the top of the panels. Animals were sampled weekly and each dot represents one observation. Linear models of the y values and 95% confidence intervals are shown as lines or shaded areas, respectively. The green lines represent animals on the 20 ppm diet. Orange lines represent animals on the 10 ppm diet and blue lines represent animals on the 5 ppm diet. The dotted line is the mean value for adult C57BL/6 mice.

We have previously shown that both wild type and SLC48A1-deficient mice become severely anemic when placed on a diet containing ~2 ppm iron (standard mouse diets contain ~400 ppm iron) (Pek et al., 2019). To determine more precisely the dietary iron requirements of wild type and SLC48A1 deficient mice, we analyzed the iron dependence of +/+, +/*Slc48a1*^−^, and *Slc48a1*^−^/*Slc48a1*^−^ animals maintained on diets containing 20, 10, or 5 ppm iron. One of the three diets was introduced into the parental cage at P15, the time at which pups first eat solid food. At weaning (P21), the animals were segregated by genotype and were housed on wire grids to prevent recovery of iron by coprophagy. The animals' complete blood counts were monitored weekly beginning at weaning and extending over an 80-day period of observation. Animals of all three genotypes, +/+, +/*Slc48a1*^−^, and *Slc48a1*^−^/*Slc48a1*^−^, demonstrated the typical mild anemia of the post-weaning period (http://www.informatics.jax.org/greenbook/frames/frame17.shtml). On the 20 ppm diet, the red cell indices of animals of all three genotypes increased to normal levels over the course of observation (Figure 3 and Supplementary Figure 3). On the 10 ppm diet the red blood cell counts (RBC), hemoglobin (Hb; Figure 3) and hematocrit (Supplementary Figure 3) of animals of all three genotypes increased to normal levels, but the mean cell volume (MCV) of +/+ and +/*Slc48a1*^−^ mice remained at the post-weaning levels and did not increase. On the 10 ppm diet the MCV of *Slc48a1*^−^/*Slc48a1*^−^ mice decreased, indicating iron deficiency. We conclude that animals of all three genotypes become sensitive to dietary iron restriction at 10 ppm, and that the 10 ppm diet is not sufficient to sustain erythropoiesis in *Slc48a1*^−^/*Slc48a1*^−^ mice (Supplementary Figure 3). On the 5 ppm diet, the RBC of +/+ and +/*Slc48a1*^−^ mice increased to normal levels (Figure 3), but *Slc48a1*^−^/*Slc48a1*^−^ mice became severely anemic. The low post-weaning Hb levels persisted throughout the course of observation in +/+ and +/*Slc48a1*^−^ mice while the Hb levels of *Slc48a1*^−^/*Slc48a1*^−^ decreased (Figure 3). The MCV of +/+ and +/*Slc48a1*^−^ mice on the 5 ppm diet decreased while the MCV of *Slc48a1*^−^/*Slc48a1*^−^ mice increased due to the severe anemia and reticulocytosis (Supplementary Figure 3). Finally, on a 5 ppm diet the hematocrits of +/+ and +/*Slc48a1*^−^ mice stayed at the post-weaning levels and were severely decreased in *Slc48a1*^−^/*Slc48a1*^−^ mice. We conclude that the sequestering of heme as hemozoin in the RES macrophage phagolysomes In SLC48A1 deficient mice is responsible for the progressive anemia.

## Discussion

Prior to our discovery of hemozoin in SLC48A1-deficient RES macrophages, hemozoin had only been observed in the lysosomal-like organelles of blood-feeding organisms that digest hemoglobin such as malaria parasites of the genus *Plasmodium* (Francis et al., 1997; Egan, 2008; Pek et al., 2019). A search of the data available on the UCSC genome browser (https://genome.ucsc.edu/cgi-bin/hgGateway) revealed that *Plasmodium* sp. and other blood feeding parasites have no orthologs of *SLC48A1* to import heme from the lysosomes to the cytoplasm and hence would be expected to be sensitive to heme toxicity. Biochemically, the sequestering of heme in the form of non-toxic hemozoin allows the parasite to avoid heme toxicity (Schwarzer et al., 1993; Basilico et al., 2003). In cell-free systems, heme crystallization into hemozoin has been shown to be a pH and concentration-dependent reaction (Chong and Sullivan, 2003; Huy et al., 2006; Stiebler et al., 2010). In the acidic-environment of the lysosome, heme has been proposed to crystallize into hemozoin after a critical concentration has been reached (Chong and Sullivan, 2003; Stiebler et al., 2010). Our observation that hemozoin does not accumulate in the reticuloendothelial tissues of prenatal and early post-natal mice indicates the critical concentration of heme in the lysosomes of RES macrophages of SLC48A1 deficient mice is not attained until ~8 days of age (P8).

The number of erythrocytes in the post-natal mouse increases 15–20-fold in the first 28 days of life (http://www.informatics.jax.org/greenbook/frames/frame17.shtml), while the mass of the animal increases 10-fold. We propose that during the first 8 days of life, the iron needed to generate heme and hemoglobin comes mainly from maternal sources. Using the presence of hemozoin in SLC48A1-deficient mice as an indicator of erythrocyte recycling, we propose that significant erythrocyte recycling begins at approximately P8. This time-point is ~17 days after the first definitive erythrocytes enter the circulation from the fetal liver (Craig and Russell, 1964). Since the life-span of adult mouse erythrocytes has been measured between 33 and 60 days (Horký et al., 1978; Beutler, 2005; Wang et al., 2010), 17 days is consistent with detectable erythrocyte recycling beginning at 1/3–1/2 of the life span of the earliest erythrocytes.

The inability to recycle heme caused by SLC48A1 deficiency predicts that SLC48A1-deficient neonatal and adolescent mice would become increasingly dependent on dietary iron. Our data indicate that a diet of 20 ppm iron is sufficient to maintain mouse erythropoiesis, even in the absence of iron from recycled erythrocytes in SLC48A1-deficient mice. On a 10 ppm iron diet, wildtype and +/*Slc48a1* mice can supply the necessary iron for erythropoiesis, although they show signs of mild anemia. In SLC48A1-deficient mice on a 10 ppm iron diet we observed a progressive anemia that first becomes significant after 45 days on a low-iron diet. This would represent a full erythrocyte life span for those erythrocytes present at birth and the halfway point for erythrocytes present at 21 days when maternal dietary iron is no longer available (Horký et al., 1978; Beutler, 2005; Wang et al., 2010). We conclude that iron recovered from recycled red blood cells is equivalent to feeding ~10 ppm of dietary iron.

To date no genetic variants in the human *SLC48A1* gene have been associated with anemia or any other disease in humans. The SLC48A1-mediated transport of heme has been shown to be dependent on several highly conserved amino acids in the membrane-spanning domains (Yuan et al., 2012; Korolnek et al., 2014; Marciano et al., 2015). We predict that, particularly in areas of the world with iron-poor diets, idiopathic anemia may be caused by *SLC48A1* variants. In regions where dietary iron is not limiting, we predict that variants in the *SLC48A1* gene could lead to iron loading in RES macrophages, as has been described for Bantu siderosis or African Iron Overload (AIO) (Walker and Arvidsson, 1950, 1953; Bothwell, 1964; Gordeuk, 2002; Camaschella, 2015; Liu et al., 2016).

## Data Availability Statement

The datasets presented in this study can be found in online repositories. The names of the repository/repositories and accession number(s) can be found in the article/Supplementary Material.

## Ethics Statement

All animal protocols were approved by the NHGRI Animal Care and Use Committee and the Institutional Animal Care and Use Committee at the University of Maryland, College Park.

## Author Contributions

IH and DB designed the experiments and edited the manuscript. LG generated the mutant mice. JJ performed the initial genotyping. WS, LW, JT, and RP performed the experiments and wrote the manuscript. All authors contributed to the article and approved the submitted version.

## Conflict of Interest

IH is the President and Founder of Rakta Therapeutics Inc. (College Park, MD), a company involved in the development of heme transporter-related diagnostics. RP is currently employed by the company BioHealth Innovation Inc., Rockville, MD 20850. The work in this paper was all conducted before RP joined BioHealth. The remaining authors declare that the research was conducted in the absence of any commercial or financial relationships that could be construed as a potential conflict of interest.
