# Supplementary figures and images for: Normal Iron Homeostasis Requires the Transporter SLC48A1 for Efficient Heme-Iron Recycling in Mammals
Source: Front Genome Ed. 2020 Oct 20;2:8. doi: 10.3389/fgeed.2020.00008 (PMC8525403; doi:10.3389/fgeed.2020.00008)

# Supplemental Figure 1

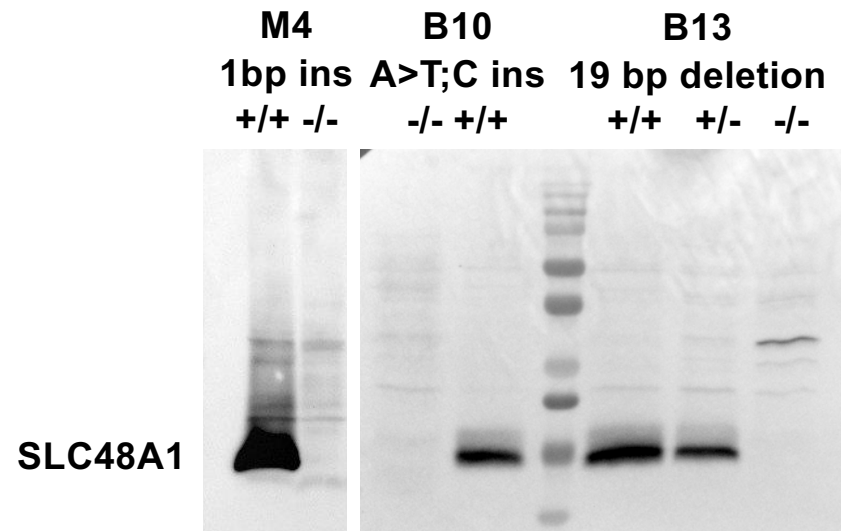

Supplement: Supplementary file 1 [file Image_1.pdf]

**Supplemental Figure 2**

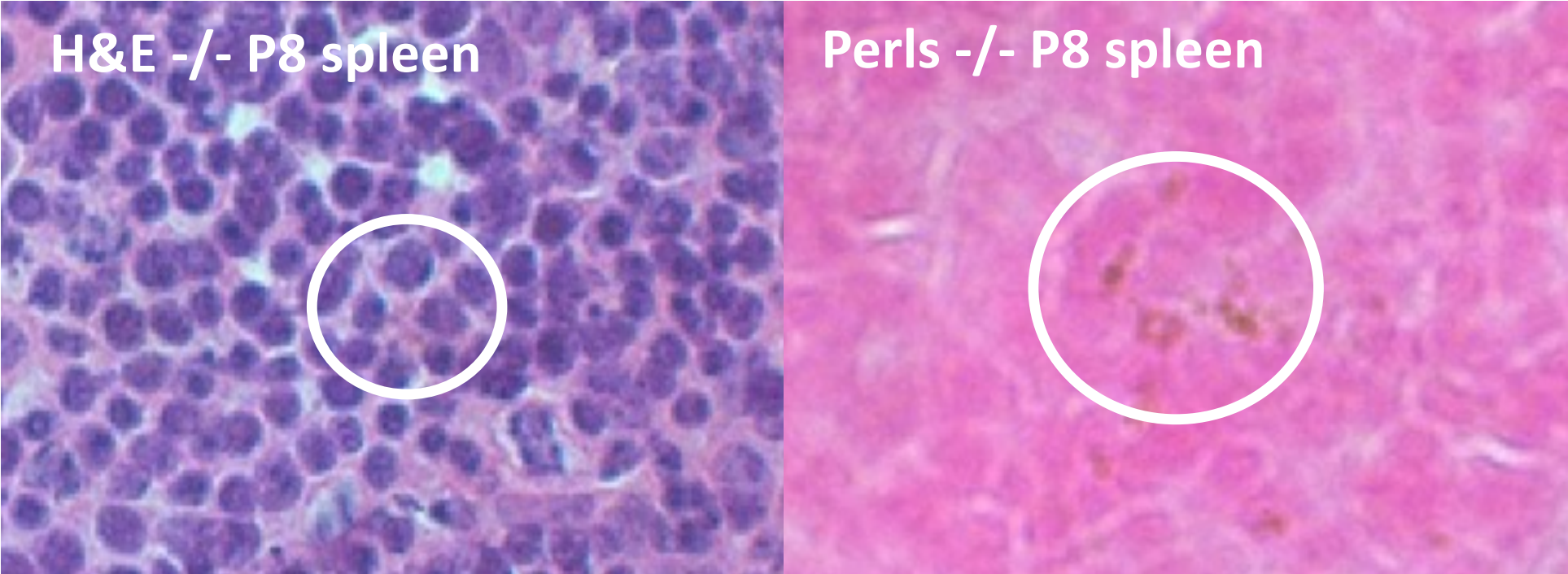

Supplement: Supplementary file 2 [file Image_2.pdf]

## Supplemental Figure 3

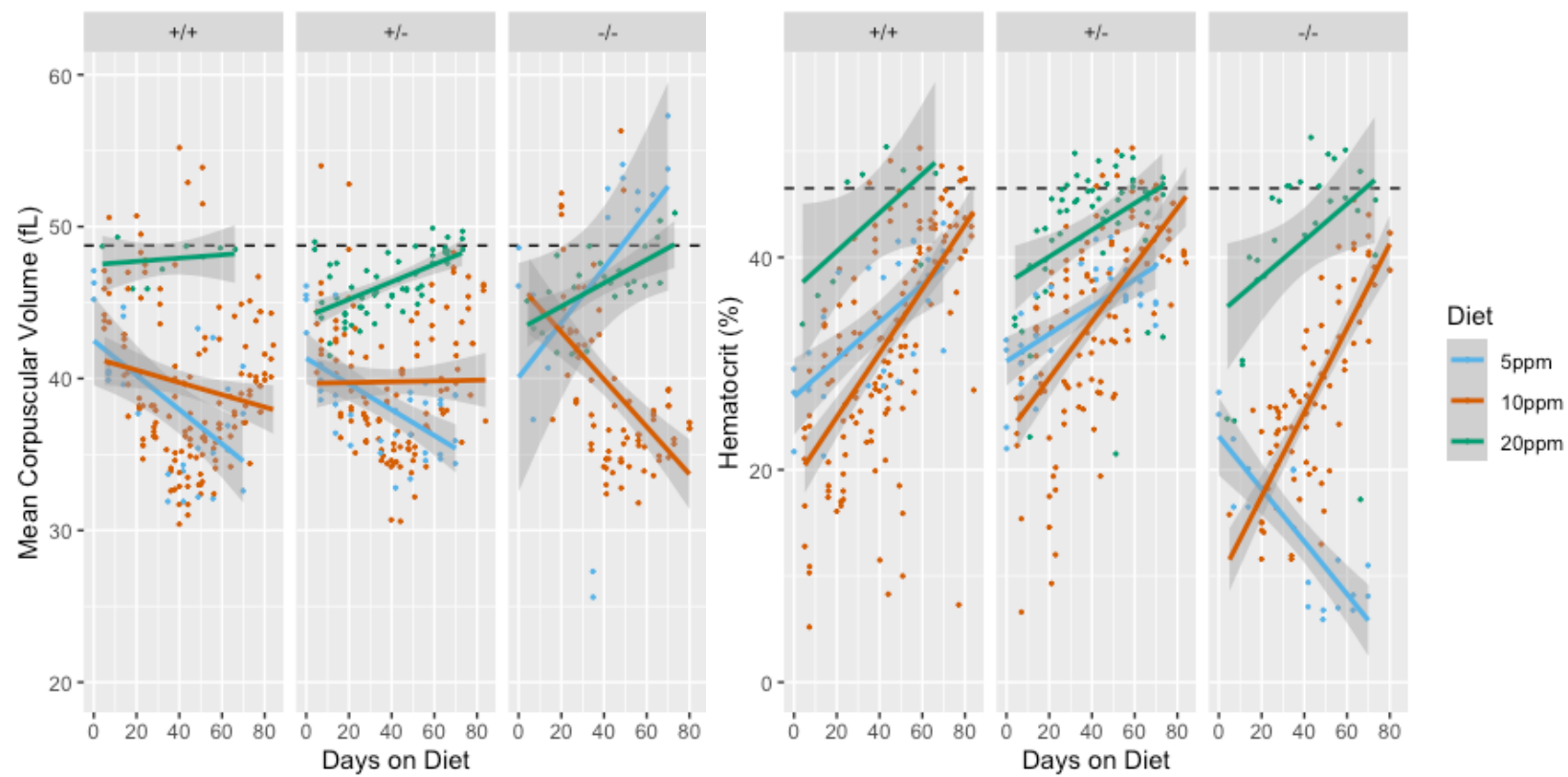

Supplement: Supplementary file 3 [file Image_3.pdf]
